# Hemolytic Anemia Due to Gamma-Glutamylcysteine Synthetase Deficiency: A Rare Novel Case in an Arab-Muslim Israeli Child

**DOI:** 10.3390/hematolrep17020020

**Published:** 2025-04-15

**Authors:** Motti Haimi, Jamal Mahamid

**Affiliations:** 1 Health Systems Managment Department, The Max Stern Yezreel Valley College, D.N. Emek Yezreel 1930600, Israel; 2 Rappaport Faculty of Medicine, Technion-Israel Institute of Technology, Haifa 3109601, Israel; 3 Meuhedet Health Services, North District, Haifa 3109601, Israel

**Keywords:** gamma-glutamylcysteine synthetase deficiency, hemolytic anemia, glutathione synthesis disorder, autosomal recessive, Arab-Muslim, consanguinity, enzymopathy

## Abstract

Background: Gamma-glutamylcysteine synthetase catalyzes the first and rate-limiting step in the synthesis of glutathione. Gamma-glutamylcysteine synthetase deficiency is a very rare condition that has so far been detected so far in nine patients from seven families worldwide. The inheritance of this disorder is autosomal recessive. Methods: We report a case of 4.11-year-old boy, of Arab-Muslim origin, living in an Arab town in Israel who presented at the age of 2 days with severe anemia, reticulocytosis, and leukocytosis. Investigation for common causes of hemolytic anemia was negative (peripheral blood smear was normal, and he had a negative Coombs test, normal G6PD, and normal flow cytometry spherocytosis). The anemia worsened during the following days (hemoglobin (Hb): 7.2 g/dL) and he needed several blood transfusions. NGS (next-generation sequencing) gene panel analysis was performed. Results: In an NGS gene panel analysis for hereditary hemolytic anemias, we found a homozygotic change in the GCLC gene—G53.385.643c379C > T(homo)pArg127Cys—which confirms the diagnosis of gamma-glutamylcysteine synthetase deficiency. An additional rare change was found in this case in the GCLC gene, with unknown clinical significance: g.53373917, c 828 + 3A > G. Except for chronic anemia (Hb levels around 8 g/dL), the child has normal physical and neurological development. Conclusions: This study reports a rare case of gamma-glutamylcysteine synthetase deficiency in a 4.11-year-old Arab-Muslim boy from Israel who presented with severe anemia at 2 days old, aiming to document the first such case in the Middle East and contribute to the medical literature on this extremely rare condition that has only been detected in nine patients worldwide. Genetic analysis revealed a homozygotic change in the GCLC gene, confirming the diagnosis, and while the patient experiences chronic anemia, he maintains normal physical and neurological development, adding valuable insights to the understanding of this rare genetic disorder. An additional rare change was found in this case in the GCLC gene, with unknown clinical significance: g.53373917, c 828 + 3A > G.

## 1. Introduction

Glutathione (L-glutamyl-L-cysteinyl-glycine, GSH) is an essential intracellular antioxidant tripeptide which is required to shield cells against endogenous and foreign poisons, free radicals, peroxides, and oxygen intermediates.

Glutathione (GSH) is essential for every cell’s metabolism. It is the major sulfhydryl compound of erythrocytes. It is transported out of cells and resynthesized, giving a turnover half-time of approximately 3 days. Glutamic acid, cysteine, and glycine make up the tripeptide known as GSH, which is produced in two successive steps. Glutathione synthetase (GS) catalyzes the second, while γ-glutamylcysteine synthetase catalyzes the first [1,2].

**Figure 1:**
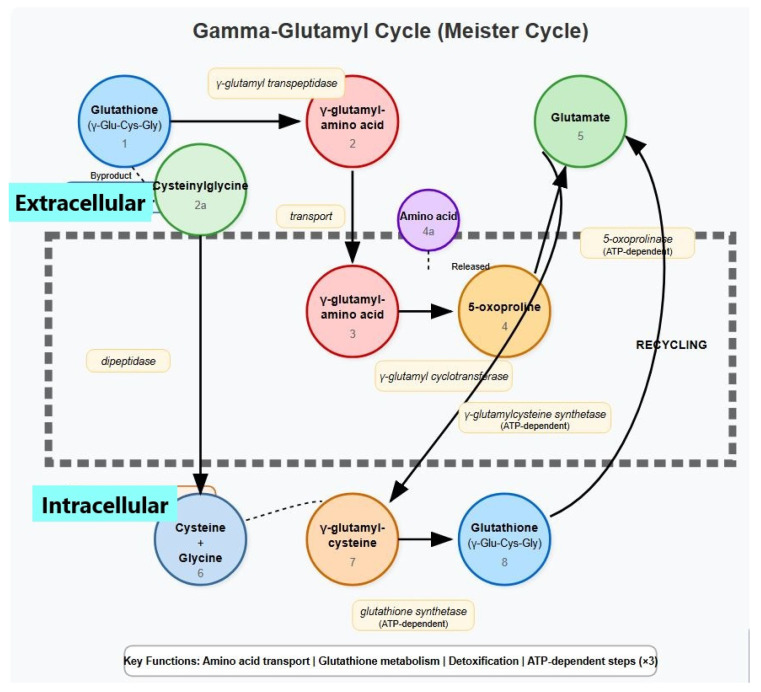
The gamma-glutamyl cycle (also known as the Meister cycle).

The gamma-glutamyl cycle (also known as the Meister cycle) is a metabolic pathway that facilitates amino acid transport across cell membranes and is involved in glutathione metabolism, as presented in Figure 1.

The enzyme γ-glutamylcysteine synthetase, which catalyzes the first step in glutathione synthesis, consists of two subunits, heavy and light, with the heavy subunit serving as the catalytic subunit [1,2,3].

One of the four disorders affecting gamma-glutamyl cycle enzymes is **gamma-glutamylcysteine synthetase deficiency**. The other three conditions are gamma-glutamyl transpeptidase insufficiency; 5-oxoprolinuria, a severe or widespread form of glutathione synthetase deficiency; and glutathione synthetase deficiency. Hemolytic anemia is associated with all but gamma-glutamyl transpeptidase deficiency. Spinocerebellar degeneration, ataxic gait, and speech impairment are among the progressive neurologic impairments that some gamma-glutamylcysteine synthetase deficiency patients may have [1,2,3,4,5,6].

Glutathione synthetase deficiency has been reported in many cases, and in certain ones, molecular lesions have been found. Conversely, γ-GCS deficiency is quite uncommon. There have been reports of 41 unrelated patients diagnosed with a hereditary deficiency of GS, which has been proved to be an autosomal recessive condition. Its severe form is characterized by central nervous system damage, hemolytic anemia, and metabolic acidosis with significant 5-oxoproline (5-oxoprolinuria) excretion in the urine [3,4,5].

A homozygous mutation in the GCLC gene (OMIM 606857), which codes for gamma-glutamylcysteine synthetase, on chromosome 6p12 results in congenital nonspherocytic hemolytic anemia-7 (CNSHA7) [6].

A thorough search of the literature was conducted using PubMed, Google Scholar, OMIM, and Orphanet, using the following words: gamma-glutamylcysteine synthetase deficiency, hemolytic anemia, congenital, and nonspherocytic hemolytic anemia.

Only nine unrelated families have been found to have hereditary GCS deficiency, a highly uncommon autosomal recessive enzymopathy [7,8,9,10,11,12].

## 2. Case Report

M.S. is a 4.11-year-old boy of Arab-Muslim origin, living in Arara, an Arab town in central Israel.

The parents are first-degree cousins. His mother is a 28-year-old Muslim with a history of beta-thalassemia minor but who is otherwise healthy. His father is 32 years old and healthy. He has a 2-year-old healthy brother.

M.S. was born after 38 weeks’ gestation by vaginal delivery (birth weight, 3050 g; length, 50 cm; head circumference, 38 cm; Apgar score 9/10). The physical examination was normal. There were no dysmorphological features.

At the age of 2 days, he presented with sever pallor. His blood count demonstrated severe anemia (Hb 10.9 g/dL), MCV-112.6 fL, reticulocytosis (19%), and leukocytosis (29,000/μL). His platelets were normal (356,000/μL), with levels of bilirubin of 5 mg/dL, creatinine of 1.2 mg/dL, urea of 35 mg/dL, AST of 112 (U/L), and ALT of 22 (U/L). Blood gases were normal (CO_2_, 52 mmol/L; pH, 7.3; bicarbonate, 21 mEq/L; lactate, 4 mmol/L).

Peripheral blood smear was normal. His blood type was A positive. His maternal blood type was O positive, and direct and indirect Coombs tests were negative.

G6PD was normal, coagulation functions were normal, and blood smears were negative.

Hemoglobin electrophoresis demonstrated an HBF value of 84% an HBA value of 15% (compatible with his age).

His flow cytometry spherocytosis test was normal. His heart echocardiogram, brain ultrasound (US), and abdominal (adrenal) US were all normal.

In the event that severe hemolytic anemia produces hypoxia, and in cases where hemolytic anemia is linked to disorders that exhibit neurological signs, the purpose of brain US is to rule out neurological consequences. The rationale behind conducting an adrenal US in this instance was that hemolytic crises, especially in neonates, can result in adrenal hemorrhage, and in cases where hemolytic anemia is thought to be part of a more broader endocrine or metabolic condition, the procedure is performed to rule out adrenal pathology in specific congenital hemolytic anemias with multi-system involvement, as well as if there are worries whether adrenal insufficiency may be causing or contributing to severe hemolytic episodes.

At the age of 5 days, he demonstrated hemoglobin levels of 7.2 g/dL, with marked reticulocytosis (5.34%), and he needed a blood transfusion (one unit). He received additional blood transfusions at the ages of 3 weeks (Hb, 7 g/dL) and 8 weeks (Hb, 6.6 g/dL).

**Figure 2:**
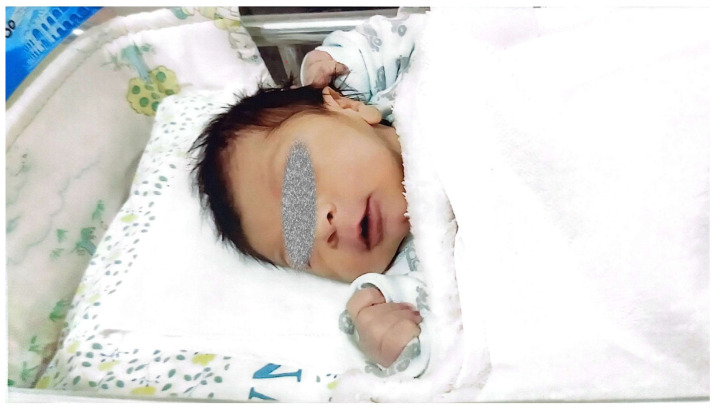
The patient at the age of 5 days.

The patient photo at the age of 5 days, showing normal morphological features, is presented in Figure 2.

At the age of 13 months, we performed genetic analysis: in an NGS (next-generation sequencing) gene panel analysis for hereditary hemolytic anemias, we performed “10070-Hemolytic Inherited anemia”, an NGS panel for hereditary hemolytic anemia including membranopathies (spherocytosis, elliptocytosis, xerocytosis, stomatocytosis, pyropoikilocytosis) and enzymopathies.

We found a homozygotic change in the GCLC gene—G53.385.643c379C > T(homo)pArg127Cys—which confirms the diagnosis of gamma-glutamylcysteine synthetase deficiency.

The homozygous mutation G53.385.643c379C > T in the GCLC gene resulting in the amino acid change pArg127Cys (arginine to cysteine at position 127) is very rare (0.0009% in the general population) and indeed consistent with gamma-glutamylcysteine synthetase deficiency. The amino acid substitution of arginine with cysteine is biochemically significant because these amino acids have very different properties.

Arginine is positively charged and basic, and has a large side chain; cysteine is neutral, contains sulfur, and can form disulfide bonds. This substitution likely disrupts the structure and function of the gamma-glutamylcysteine synthetase enzyme, which is crucial for glutathione synthesis. Without sufficient glutathione, red blood cells become vulnerable to oxidative damage, leading to the hemolytic anemia observed, as observed in our patient.

An additional rare change was found in this case in the *GCLC* gene, with unknown clinical significance: g.53373917, c 828 + 3A > G. There were no signs of other changes associated with hemolytic anemias.

Our patient needed two additional blood transfusions at the ages of 3 years (before adenoidectomy) and 4 years (before a dental procedure (tooth extraction)).

Except for chronic anemia (Hb levels around 8 g/dL), the child has normal physical and neurological development. On physical examination, including neurologic assessment, no abnormalities were found.

He is in the 57th percentile of weight and in the 64th percentile of height. He receives folic acid regularly (2.5 mg/day).

His recent lab results are as follows: Hb, 8.1 g/dL; RBC, 27.4 (M/mm3); MCV, 82 fL; reticulocytes, 3.1%. He only receives blood transfusions occasionally when necessary.

Table 1 displays the patient’s treatment history and evaluation.

## 3. Discussion

Only nine unrelated families have been found to have a hereditary *GCS* deficiency, a highly uncommon autosomal recessive enzymopathy [7,8,9,10,11,12].

Although recurrent episodes of anemia and jaundice are the most prevalent clinical symptoms of *GCS* deficiency, two cases have been linked in the past to severe neurological abnormalities [7,13], and one case has been linked to intellectual disability [11].

Single-point mutations in the *GCS* gene’s coding sequence have been found to be the underlying etiology of *GCS* deficiency in the two most recently reported cases of this disorder [11,12].

In a patient of Moroccan descent, M. Mañú Pereira et al. [14] described a new mutation, a single C > T transversion at cDNA nucleotide 1241 in the *g-GCS* gene. This is the fourth instance of *GCS* deficiency that has been reported to date where mental impairment and severe neuropathy are both associated with chronic anemia.

Historically, the earliest documented cases of glutathione deficiency in red blood cells came from a Dutch family studied in the 1960s. In this family, which had consanguineous parents, five cases were identified across two groups of siblings. All affected individuals had very low glutathione levels (less than 10% of normal) and showed signs of nonspherocytic hemolytic anemia, a group of conditions for which the main feature is the premature destruction of red blood cells, usually due to a deficiency of vital enzymes required for glycolysis and red blood cell nucleotide metabolism. “Nonspherocytic” means the red blood cells are not sphere-shaped like normal red blood cells. Their blood also showed reduced glyoxalase activity, which makes sense since this enzyme needs glutathione to function [15,16].

The first documented cases of gamma-glutamylcysteine synthetase (GCS) deficiency were reported in 1972 by Konrad et al. [7]. They identified a German brother and sister with a slightly different condition—they lacked the enzyme that performs the first step in making glutathione (gamma-glutamylcysteine synthetase). Unlike the Dutch family, their parents were not related. What made their case particularly interesting was that they also developed neurological problems, specifically spinocerebellar degeneration, later in life. People who carried just one copy of the defective gene (heterozygotes) had medium levels of the enzyme but normal glutathione levels in their red blood cells. Both affected sibs had late-onset spinocerebellar degeneration.

The same sibs were reported by Richards et al. in 1974 [13]. Their key clinical features included the following: relatively well-compensated anemia, progressive spinocerebellar degeneration, extremely low erythrocyte glutathione (less than 5% of normal), severely reduced GCS activity (2–7% of normal), normal glutathione synthetase (GS) activity, learning disabilities with dyslexia, severe progressive ataxia with myopathy, and psychotic episodes (possibly triggered by sulfamethoxazole–trimethoprim). In 2003, Richards et al. identified the underlying genetic cause: a 379C > T mutation resulting in an Arg127Cys amino acid change [13].

After these initial cases, six additional cases were reported: two by Beutler et al. (in 1990 and 1999) [8,10], two by Hirono et al. (1996) [9], and two by Ristoff et al. (2000) [11]. Most patients showed moderate-to-severe hemolytic anemia that either appeared during the neonatal period or early childhood and persisted throughout life or manifested as compensated hemolysis with recurring episodes of anemia and jaundice:

- In 1990, Beutler et al. [8] found a second family with the same enzyme (gamma-glutamylcysteine synthetase) deficiency. Importantly, these patients did not have any neurological symptoms, showing that nervous system problems are not always part of this condition. This family was also notable because researchers identified a specific mutation in their GCLC gene.
- By 1996, the first Japanese cases were reported by Hirono et al. (1996) [9]: three unrelated patients with chronic nonspherocytic hemolytic anemia and severely low red blood cell glutathione. Two of these patients had a moderate deficiency of gamma-glutamylcysteine synthetase, while one had a deficiency of a different enzyme, glutathione synthetase.
- Ristoff et al. (2000) [11] described a 68-year-old lady who had compensated hemolytic anemia and a history of temporary jaundice. Her eleven-year-old grandson, who had a history of neonatal jaundice, was similarly GCS-deficient. GCS activity in the erythrocytes of both individuals was less than 2% of normal, confirming the enzyme deficiency.

In 2007, M. Mañú Pereira et al. [14] reported a previously undescribed mutation of hereditary g-glutamylcysteine synthetase (GCS) deficiency, which was found in a 5-year-old boy of Moroccan origin. He presented with chronic hemolytic anemia, delayed psychomotor development, and progressive motor sensitive neuropathy of the lower extremities. This was the fourth case of GCS deficiency presenting neuropathy.

Table 2 shows the nine cases of gamma-glutamylcysteine synthetase deficiency reported so far in the literature.

## 4. Summary and Conclusions

Hereditary gamma-glutamylcysteine synthetase (GCS) deficiency is an extremely rare autosomal recessive disorder documented in only nine unrelated families worldwide. While recurrent anemia and jaundice are the primary symptoms, some cases present with neurological complications.

The condition was first identified in a Dutch family in the 1960s, with the first confirmed *GCS* deficiency documented in 1972 in German siblings who later developed spinocerebellar degeneration. Subsequent cases were reported by various researchers (Beutler, Hirono, Ristoff) between 1990 and 2000, showing varying clinical presentations.

A 2007 case involving a Moroccan boy represented the fourth documented instance where GCS deficiency presented with both chronic anemia and neurological manifestations (developmental delay and peripheral neuropathy). Most cases involve a specific mutation in the GCLC gene, with the 379C > T mutation (Arg127Cys) being identified in some patients.

**What is particularly interesting about this case is the following:**

- The mutation found (379C > T) is the same as that identified in the original German siblings from 1972.
- Unlike those cases, our patient shows no neurological symptoms, but close surveillance is needed, since these symptoms may appear at later stages.
- The presence of consanguinity aligns with autosomal recessive inheritance.
- The patient has achieved relatively good compensation of his anemia, requiring transfusions mainly for surgical procedures rather than routine management.

**The key takeaways from this case report and disorder are as follows:**

1. **Extreme Rarity**: Hereditary *GCS* deficiency is an autosomal recessive disorder documented in only nine families worldwide (our case is the tenth).
2. **Primary Symptoms**: The condition primarily presents with recurrent anemia and jaundice, with some cases showing neurological complications.
3. **Genetic Basis**: Most cases involve mutations in the *GCLC* gene, with the 379C > T mutation (Arg127Cys) identified in several patients.
4. **Consanguinity Risk**: The Israeli case highlights the increased risk of rare recessive disorders in consanguineous marriages.
5. **Variable Presentation**: The severity and specific symptoms can vary significantly between cases, from purely hematological symptoms to those with neurological manifestations.
6. **Case Example**: The newly described case from Israel involved a child born to consanguineous parents who presented with severe anemia requiring multiple transfusions initially but now manages the condition with daily folic acid and only occasional transfusions.
7. **Potential for Normal Development**: The Israeli child is currently exhibiting normal neurological and physical development despite the severe initial presentation, indicating that affected individuals may flourish with appropriate therapy, even though neurological signs may develop later.

## Figures and Tables

**Table 1:** Timeline summary.

Event	Date	Treatment/Investigation
Date of birth	April 2020	
Diagnosis of hemolytic anemia	Age of 2 days (April 2020)	Investigation for hemolytic anemia
Age of 5 days	April 2020	Blood transfusion
Age of 3 weeks	May 2020	Blood transfusion
Age of 8 weeks	June 2020	Blood transfusion
Genetic diagnosis	Age of 13 months (May 2021)	NGS gene panel analysis
Adenoidectomy	April 2024	Blood transfusion
Dental procedure	July 2024	Blood transfusion
Since birth		Hematologic follow-up

**Table 2:** Summary of reported cases of gamma-glutamylcysteine synthetase (GCS) deficiency.

Name of Author	Year	Patients	Reference
**Konrad et al.** **Richards et al.**	1972 1974	Two German patients: a brother and sister	[7,13]
**Beutler**	1990	A 22-year-old Polish woman and her mother	[8,10]
**Hirono et al.**	1996	Three Japanese patients	[9]
**Ristoff et al.**	2000	Two patients: a 68-year-old Dutch woman and her 11-year-old grandson	[11]
**Mañú Pereira et al.**	2007	One patient: a 5-year-old Moroccan child	[14]

## Data Availability

The data presented in this study are available on request from the corresponding author due to privacy reasons.
